# Targeting KDM1A in Neuroblastoma with NCL-1 Induces a Less Aggressive Phenotype and Suppresses Angiogenesis

**DOI:** 10.3390/jcm13206081

**Published:** 2024-10-12

**Authors:** Annika Sprüssel, Takayoshi Suzuki, Naoki Miyata, Kathy Astrahantseff, Annabell Szymansky, Joern Toedling, Theresa M. Thole-Kliesch, Annika Ballagee, Marco Lodrini, Annette Künkele, Matthias Truss, Lukas C. Heukamp, Susanne Mathia, Falk Hertwig, Christian Rosenberger, Angelika Eggert, Hedwig E. Deubzer, Johannes H. Schulte

**Affiliations:** 1 Department of Pediatric Oncology and Hematology, Charité—Universitätsmedizin Berlin, Campus Virchow Klinikum, Augustenburger Platz 1, 13353 Berlin, Germanykathy.astrahantseff@charite.de (K.A.); annabell.szymansky@charite.de (A.S.); theresa.thole@charite.de (T.M.T.-K.); marco.lodrini@charite.de (M.L.); annette.kuenkele@charite.de (A.K.); matthias.truss@charite.de (M.T.); angelika.eggert@charite.de (A.E.); hedwig.deubzer@charite.de (H.E.D.); 2 German Cancer Consortium (DKTK), Partner Sites Berlin and Tuebingen, German Cancer Research Center (DKFZ), Im Neuenheimer Feld 280, 69120 Heidelberg, Germany; 3 SANKEN, Osaka University, 8-1 Mihogaoka, Ibaraki 567-0047, Osaka, Japan; suzukit@koto.kpu-m.ac.jp; 4 CREST, Japan Science and Technology Agency (JST), Honcho 4-1-8, Kawaguchi 332-0012, Saitama, Japan; 5 Graduate School of Pharmaceutical Sciences, Nagoya City University, 3-1, Tanabe-dori, Mizuho-ku, Nagoya 467-8603, Aichi, Japan; yakka@phar.nagoya-cu.ac.jp; 6 Berlin Institute of Health (BIH), Anna-Louisa-Karsch-Straße 2, 10178 Berlin, Germany; 7 Hematopathology Hamburg, Fangdieckstraße 75A, 22547 Hamburg, Germany; heukamp@hp-hamburg.de; 8 Department of Vegetative Physiology, Charité—Universitätsmedizin Berlin, Charitéplatz 1, 10117 Berlin, Germany; susanne.mathia@bih-charite.de; 9 Department of Nephrology, Charité—University Medicine Berlin, Charitéplatz 1, 10117 Berlin, Germany; christian.rosenberger@charite.de; 10 Experimental and Clinical Research Center (ECRC) of Charité and Max-Delbrück-Center of Molecular Medicine in the Helmholtz Association, Lindenberger Weg 80, 13125 Berlin, Germany; 11 Max-Delbrück Center of Molecular Medicine in the Helmholtz Association, Robert-Rössle-Straße 10, 13125 Berlin, Germany; 12 Department of Pediatric Hematology and Oncology, University of Tuebingen, Hoppe-Seyler-Straße 1, 72076 Tuebingen, Germany

**Keywords:** epigenetics, histone demethylase, LSD1, targeted therapy, pediatric cancer

## Abstract

**Background:** The KDM1A histone demethylase regulates the cellular balance between proliferation and differentiation, and is often deregulated in human cancers including the childhood tumor neuroblastoma. We previously showed that KDM1A is strongly expressed in undifferentiated neuroblastomas and correlates with poor patient prognosis, suggesting a possible clinical benefit from targeting KDM1A. **Methods:** Here, we tested the efficacy of NCL-1, a small molecule specifically inhibiting KDM1A in preclinical models for neuroblastoma. **Results:** NCL-1 mimicked the effects of siRNA-mediated KDM1A knockdown and effectively inhibited KDM1A activity in four neuroblastoma cell lines and a patient-representative cell model. KDM1A inhibition shifted the aggressive tumor cell phenotypes towards less aggressive phenotypes. The proliferation and cell viability was reduced, accompanied by the induction of markers of neuronal differentiation. Interventional NCL-1 treatment of nude mice harboring established neuroblastoma xenograft tumors reduced tumor growth and inhibited cell proliferation. Reduced vessel density and defects in blood vessel construction also resulted, and NCL-1 inhibited the growth and tube formation of HUVEC-C cells in vitro. **Conclusions**: Inhibiting KDM1A could attack aggressive neuroblastomas two-fold, by re-directing tumor cells toward a less aggressive, slower-growing phenotype and by preventing or reducing the vascular support of large tumors.

## 1. Introduction

Neuroblastoma arises from neural crest progenitor cells, and is the most common extracranial childhood tumor, accounting for approximately 15% of all childhood cancer deaths [1]. Neuroblastoma biology ranges from tumors that often spontaneously regress or differentiate with little or no treatment to highly aggressive disseminated disease that often progresses despite aggressive multimodal therapy [2]. Long-term survival occurs in <50% of patients treated with current regimens for high-risk disease, and approximately half of these cases relapse or recur [1,3], for which no effective treatment options currently exist. The identification of druggable targets in aggressive neuroblastoma and preclinical testing of targeting drugs is crucial for developing novel options that can be combined into precision treatment protocols.

KDM1A (formerly LSD1) was the first protein shown to be capable of demethylating histones [4], and has since become recognized as regulating the dynamics of several key cellular processes via its regulation of chromatin structure [5]. KDM1A regulates gene transcription directed by RNA polymerase II by removing the activation marks on histone 3, which include the mono- and dimethyl lysines 4 (H3K4) or repressive marks at H3K9 [5]. Through its regulation of both chromatin structure and gene transcription, KDM1A can regulate the epithelial–mesenchymal transition [6,7,8], affecting the invasive properties of cells, as well as shifting the balance between self-renewal and differentiation in stem cells [9]. We have previously shown that KDM1A is strongly expressed in undifferentiated neuroblastomas, and correlates with poor patient prognosis [10]. High-level KDM1A expression has also been reported in prostate [11], bladder [12], breast [13], colorectal [14], gastric [15] and lung [16] cancers as well as sarcomas [17] and acute myeloid leukemia [18]. The processes to which KDM1A activity has been linked and its prominent dysregulation in human cancers make it an attractive therapeutic target.

KDM1A is a member of the monoamine oxidase (MAO) superfamily. Tranylcypromine, which irreversibly inhibits this enzyme family via a suicide-inactivation mechanism involving covalent modification of the FAD cofactor, has limited activity against KDM1A, spurring the development of several tranylcypromine derivatives as potential KDM1A inhibitors (reviewed in [5,19]). After demonstrating specificity for KDM1A and anti-cancer and pro-differentiation activities in preclinical models (reviewed in [5]), a total of nine LSD1 inhibitors have entered clinical trials for hematological and/or solid cancers. Seven of them (tranylcypromine, iadademstat (ORY-1001), bomedemstat (IMG-7289), GSK-2879552, INCB059872, JBI-802, phenelzine) covalently bind the FAD cofactor, and two are non-covalent LSD1 inhibitors (pulrodemstat, seclidemstat) [20].

N-[(1S)-3-[3-(trans-2-aminocyclopropyl)phenoxy]-1-(benzylcarbamoyl)propyl] benzamide (NCL-1) is a mechanism-based inhibitor developed by Takayoshi Suzuki and Naoki Miyata (21), which inhibited the viability of HeLa (cervical adenocarcinoma), HCT116 (colorectal cancer), PC-1 (pancreas cancer), KYSE-150 (esophageal squamous cell carcinoma) and SH-SY5Y (neuroblastoma) cell lines [21] and demonstrated relatively strong anti-tumor activity in xenograft models of prostate cancer via the regulation of apoptosis and autophagy [22]. Here, we examined the anti-tumor activity of NCL-1 in neuroblastoma cell lines grown in culture and as xenografts in nude mice. Our analyses also examined the effects on the tumor microenvironment, especially endothelial cells and their role in tumor angiogenesis.

## 2. Materials and Methods

### 2.1. Cell Lines and Inhibition

The SH-EP, SK-N-BE, SK-N-AS and IMR-5 neuroblastoma cell lines were cultured in RPMI-1640 supplemented with 10% fetal bovine serum (FBS) and 2 mM penicillin and streptomycin (GIBCO^®^ Invitrogen by Life Technologies Corp., Carlsbad, CA, USA). The SH-EP and SK-N-AS cell lines have an *MYCN* diploid status while the SK-N-BE and IMR-5 cell lines harbor *MYCN* amplifications. Cell line identity was verified by IDEXX BioResearch company (Ludwigsburg, Germany). Human umbilical vein endothelial cells from a single donor (HUVEC-C) were purchased from PromoCell (PromoCell GmbH, Heidelberg, Germany) and cultured on 1% (*w/v*) gelatin in phosphate-buffered saline (Sigma-Aldrich, Munich, Germany) in 200PRF media supplemented with low serum growth supplement (GIBCO^®^ Invitrogen). OHC-NB1 is an *MYCN*-amplified neuroblastoma model established from a primary bone marrow metastasis that was initially maintained subcutaneously xenografted in mice to preserve the genomic clonal heterogeneity in the metastasis from which it was derived (a generous gift from T. Thole and H. Deubzer, [23]). OHC-NB1 cells grow partly in monolayer and partly as aggregates in suspension. OHC-NB1 cells were cultured in DMEM (high glucose, pyruvate) medium supplemented with 2 µg/mL heparin, 20 ng/mL EGF, and 20 ng/mL FGF2 (GIBCO^®^ Invitrogen). All cells were maintained at 37 °C under 5% CO2. NCL-1 was a gift from Naoki Miyata and Takayoshi Suzuki, and the lyophilisate was dissolved in DMSO for a stock concentration of 50 mM.

### 2.2. Xenografts in NMRI nu/nu Mice

SK-N-BE or SK-N-AS cells (1 × 10^7^ cells) were subcutaneously injected with 200 µL BD Matrigel™ (BD Biosciences, Franklin Lakes, NJ, USA) into the flanks of immunocompromised nu/nu mice to generate established xenograft tumors between 50 and 200 mm^3^ (measured daily using calipers). NCL-1 was dissolved in 18% polysorbate 80 and 10% ethanol in physiological saline (adjuvant). Mice (5 harboring SK-N-BE and 10 harboring SK-N-AS tumors) were intraperitoneally injected daily with 5 mg NCL-1 (200 µL total volume). The control cohort (5 mice harboring SK-N-BE and 11 harboring SK-N-AS tumors) were injected daily with adjuvant alone. Mice harboring 500–800 mm^3^ tumors of either SK-N-BE or SK-N-AS cells were treated twice daily with 10 mg NCL-1 (20 mg/d) or adjuvant (control group) for 3 d to achieve a higher dosage regimen over a shorter period for tumor immunohistochemistry. Animal handling and care conformed to national and EU regulatory standards and was approved by Landesamt für Natur, Umwelt und Verbraucherschutz Nordrhein-Westfalen.

### 2.3. KDM1A Activity Assay

Whole-cell lysates were prepared in RIPA buffer containing 1% (*w/v*) SDS and Complete^®^ protease inhibitor cocktail (Roche) and analyzed using the EpiQuik™ Histone Demethylase LSD1 Activity/Inhibition Assay Kit (Epigentek, New York, NY, USA) according to the manufacturer’s instructions. Technical duplicates were used in experiments repeated at least three times.

### 2.4. EC_50_ and IC_50_ Determination and Cell Viability, Proliferation and Death Analysis

Cells (8000–20,000/well) were seeded into 96-well plates for all assays. EC_50_ determinations were based on cell viability in all cell models. Viability was assessed in most cell lines using the 3-(4,5-dimethylthiazol-2-yl)-2,5-diphenyltetrazolium bromide (MTT) assay with the Roche Cell Proliferation Kit (Mannheim, Germany), and in OHC-NB1 cells and SK-N-BE cells (in experiments with retinoic acid) using the Promega CellTiter-Glo^®^ Luminescent Cell Viability Assay to prevent loss of data from cells growing in suspended aggregates. For all cells except OHC-NB1, proliferation was assessed using the colorimetric BrdU-based Cell Proliferation ELISA (Roche), and death was assessed using the Cell Death Detection ELISAPLUS (Roche). Proliferation and cell death were assessed in the OHC-NB1 cell model from cell counts (Vi-CELL XR 2.04, Beckman Coulter GmbH, Krefeld, Germany) after vital staining with trypan blue. All assays were performed according to the manufacturer’s directions. Technical duplicates were used in experiments repeated at least three times.

### 2.5. Endothelial Tubulogenesis and Wound Healing

HUVEC-C cells (cultured to 80% confluency) were trypsinized and washed in phosphate-buffered saline (PBS), before seeding 5 × 105 cells in 200 µL Matrigel™ (BD Biosciences) into 24-well plates. Culture wells were supplied with 200PRF media containing low serum growth supplement. A negative control for tubulogenesis received only 200PRF media. NCL-1 or DMSO (control) was added at plating, and photomicrographs were taken every 2 h during the 24 h monitoring period. Confluent HUVEC-C cultures in 24-well plates coated with 1% gelatin (Sigma-Aldrich) were used for wound healing assays. Linear scratches were performed every 2 h and photomicrographs taken of the same positions in every well. Tubulogenesis was analyzed using ImageJ 1.47p software (NIH, Bethesda, MD, USA).

### 2.6. Gene and Protein Expression

*NTS* (Primer Hs_NTS_1_SG; Qiagen, Hilden, Germany) and *MAP2* expression (forward 5′-TCTCTTCTTCAGCACGGCG-3′, reverse 5′-GGGTAGTGGGTGTTGAGGTACC-3′) [24] were monitored by quantitative real-time reverse transcriptase PCR (qPCR) using the QuantiTect SYBR Green PCR kit (Qiagen). Total RNA was isolated from tissue or cells using the RNeasyMicro kit (Qiagen), and cDNA synthesis was performed using the SuperScript reverse transcription kit (Invitrogen). Expression values were normalized to the housekeeping gene, GAPDH (forward 5′-CATCAAGAAGGTGGTGAAGC-3′, reverse 5′-GAGCTTGACAAAGTGGTCGT-3′), before calculating relative expression changes with the 2^−ΔΔCt^ method [25]. Technical duplicates were used in experiments repeated at least three times. MKI67, cleaved CASP3, and PECAM1 were detected immunohistochemically as previously described ([26]; antibodies in Table S1) in xenograft tumor sections subsequently stained with hematoxylin/eosin [26]. HIF1A was detected in xenograft tumor sections as previously described ([27]; antibodies in Table S1). ACTB and KDM1A expression and dimethylated H3K4 and H3K9 were detected using Western blotting (antibodies in Supplementary Table S1). Cells were extracted in RIPA buffer with 1% (*w/v*) SDS and with protease inhibitors (Complete^®^, Roche, Mannheim, Germany) and sonicated for 0.5 h (Bioruptor™ Plus, Diagenode, Liège, Belgium). Coomassie blue staining of SDS-PAGE gels and Ponceau staining of the nitrocellulose membranes were performed to visualize equal protein loading and transfer. Proteins of interest were visualized using secondary antibodies conjugated to horseradish peroxidase (GE Healthcare, Chicago, IL, USA) and chemiluminescence (ECL™ Prime Western Blotting System, GE Healthcare). ImageJ software (NIH, Bethesda, MD, USA) was used to calculate relative protein expression from bands in Western blots.

### 2.7. Assessing Neurite Outgrowth

Neurite outgrowth is a marker of neuroblastoma cell differentiation. Selective binding of phalloidin to filamentous actin (required for extension of neurite-like structures) can be microscopically assessed using fluorophore-conjugated phalloidin (AlexaFluor^®^ 555 Phalloidin, Cell Signaling Technology, Danvers, MA, USA). Cells were grown on glass coverslips for phalloidin staining, and stored for up to 1 month in PBS containing 30% sucrose at −80 °C before rinsing (PBS) and fixing in 4% paraformaldehyde. Coverslips were washed with PBS, blocked for 1 h in 5% bovine serum albumin in PBS with 0.3% TritonTM X-100, and stained overnight at 4 °C in the dark with the phalloidin conjugate (1:20 in blocking solution). Coverslips were mounted in VECTASHIELD^®^ Antifade Mounting Medium with DAPI (Novus Biologicals™, Fisher Scientific, Hampton, NH, USA) before viewing on an Olympus BX43 microscope with XC10 camera (Olympus Europa SE & Co. KG, Hamburg, Germany). Neurite-like structures were manually counted in 125 randomly selected fields from 2 slides per treatment group using the NeuronJ [28] plugin in ImageJ software (NIH, Bethesda, MD, USA).

### 2.8. Statistical Analyses

All statistical analyses were conducted using Graph Pad Prism 5.0 (GraphPad Software Inc., La Jolla, CA, USA), including EC_50_ calculations in cell lines and comparisons between in vitro or in vivo treatment groups using Student’s two-sided *t*-test. Results were considered significant if *p* < 0.05.

## 3. Results

### 3.1. NCL-1 Efficiently Suppresses Neuroblastoma Cell Viability In Vitro

**Figure 1:**
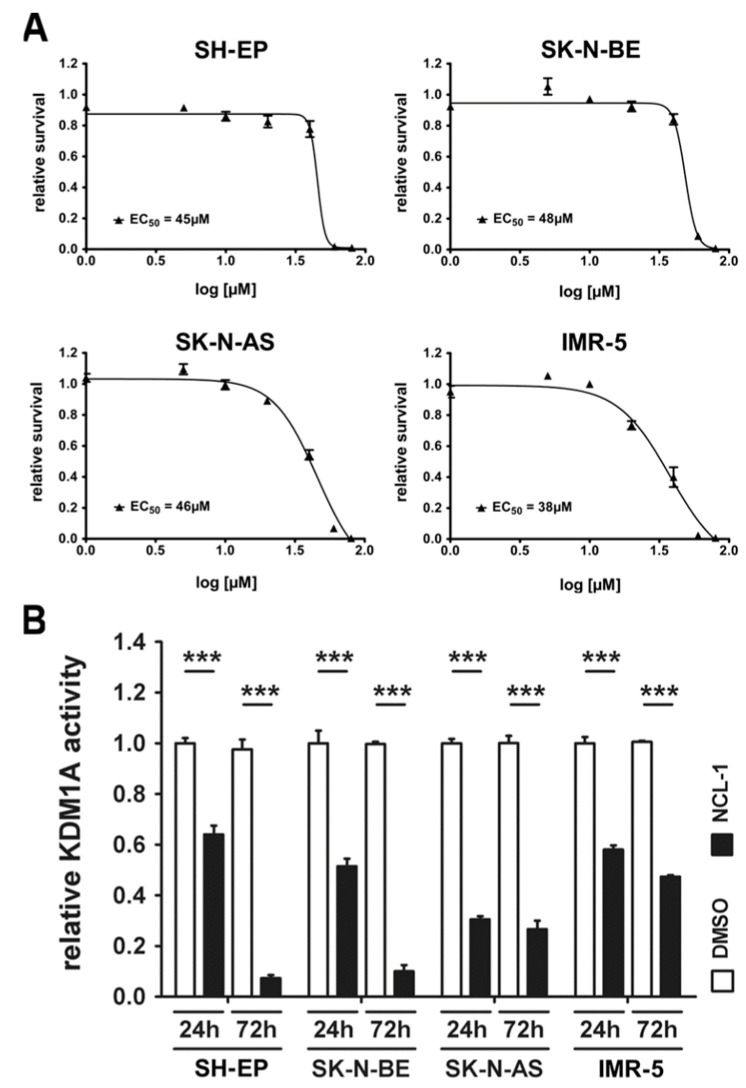
NCL-1 efficiently suppresses neuroblastoma cell viability in vitro and inhibits KDM1A-mediated histone demethylation. (**A**). The effective dose of NCL-1 required to inhibit cell viability by 50% (EC_50_) was calculated for the human neuroblastoma cell lines SH-EP, SK N BE, SK-N-AS, and IMR-5. The EC_50_ and the inhibitory concentration (IC_50_) values were identical. Cell viability was assessed using the MTT assay after treatment with 5–80 µM NCL-1 for 72 h. Treatment with the solvent (DMSO) alone served as the control. (**B**) Whole-cell lysates were prepared in RIPA buffer from cultures treated with 40 µM NCL-1 or DMSO (control) for 24 h and 72 h, then sonicated to disrupt protein complexes. Relative KDM1A activity was analyzed using the EpiQuik™ KDM1A/LSD1 activity/inhibition assay. Significant differences between treatment groups were assessed by Student’s *t*-test. All comparisons between treatment groups and the corresponding controls had *p*-values < 0.001. *** *p* < 0.0001.

We first examined the effect of NCL-1 on neuroblastoma cell viability in culture. Neuroblastoma cell lines were selected with various molecular oncogenic backgrounds to test anti-tumor activity in different high-risk scenarios. SK-N-BE and IMR-5 cells harbor *MYCN* amplifications [29,30], with SK-N-BE cells also harboring an *ALK* gain [31]. SH-EP cells harbor the activating *ALK*^F1174L^ mutation [32], and SK-N-AS cells are wildtype for these drivers but retain a high-risk phenotype [33]. The neuroblastoma cells were treated for 72 h with NCL-1 concentrations ranging from 5 to 80 µM or a DMSO solvent control, and the viability was assessed with MTT assays. The NCL-1 EC_50_ was in the range of 38–48 µM for all the neuroblastoma cell lines tested (Figure 1A). We selected 40 µM NCL-1 as the in vitro testing standard concentration based on these results, and examined KDM1A activity via ELISA. The KDM1A activity was strongly inhibited in every cell line after 24h, being almost completely suppressed in the SH-EP and SK-N-BE cells, and further suppressed in the IMR-5 cells after 72 h (Figure 1B). The KDM1A activity was not further diminished in SK-N-AS cells by longer treatment and remained at the plateau reached at 24 h (Figure 1B). The global dimethylation levels at H3K4 and H3K9 examined at 24 h and 72 h of NCL-1 treatment were strongly increased in IMR-5 cells and slightly increased in SK-N-AS cells (Supplementary Figure S1). No changes were detected in the SH-EP model (Supplementary Figure S1). The expression of ACTB used as a loading control in Western blot analysis varied in some of the conditions investigated, thus limiting the informative value (Supplementary Figure S1). The discrepancy between the cell responses most likely stems from the specificity of KDM1A inhibition by NCL-1. KDM1A acts in complexes that fine-tune methylation patterns at specific promotors and/or enhancers rather than regulating global histone methylation, which would be affected only in part by NCL-1 treatment. Our experiments demonstrate that NCL-1 effectively inhibits KDM1A and suppresses the viability of aggressive neuroblastoma cells with various molecular drivers.

### 3.2. NCL-1 Treatment Phenocopies KDM1A Knockdown In Vitro

**Figure 2:**
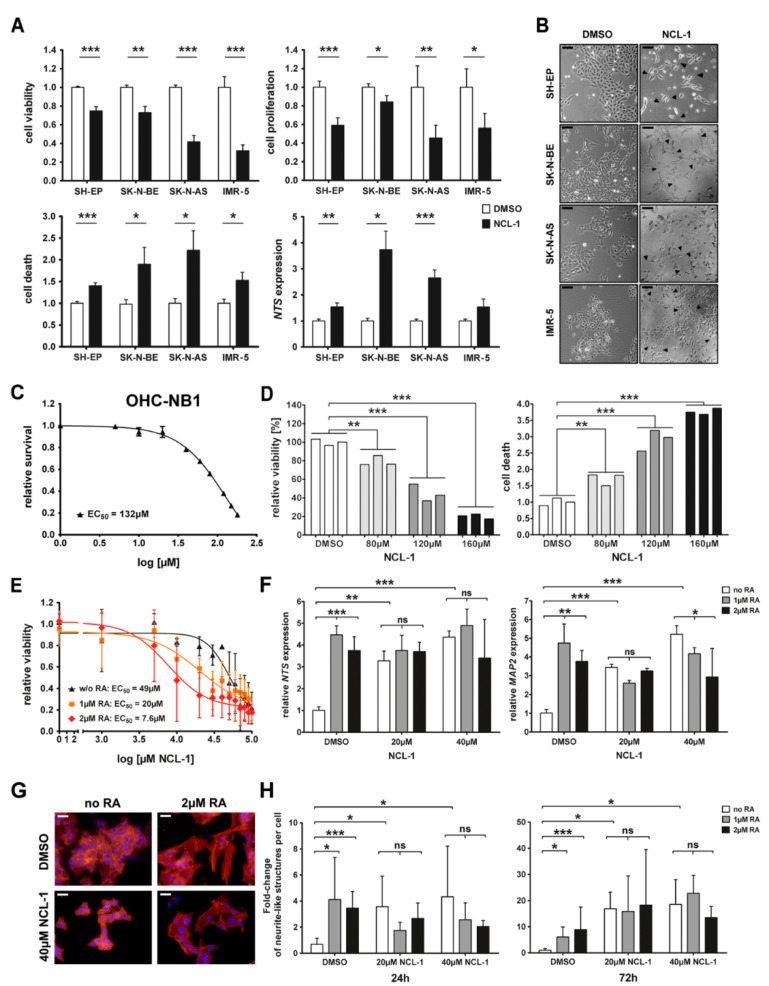
KDM1A inhibition with NCL-1 mimics functional effects of KDM1A knockdown in vitro. (**A**). Neuroblastoma cell lines were treated for 72 h with 40 µM NCL-1. Cell viability was assessed in MTT assays, cell proliferation in an ELISA (BrdU integration), cell death in the Cell Death ELISA, and NTS expression by qPCR. (**B**). Outgrowing neurites are indicated by black arrowheads in micrographs of cultures treated with 40µM NCL-1 or DMSO (control). Scale bars: 50 µm. (**C**). Cell viability (CellTiter-Glo^®^ Luminescent Cell Viability Assay) was used to assess effective dose of NCL-1 required to inhibit 50% activity (EC_50_) in the OHC-NB1 model at 72 h. The EC_50_ and the inhibitory concentration (IC_50_) values were identical. Treatment with DMSO alone served as the control. (**D**). OHC-NB1 model viability and cell death after 72 h NCL-1 treatment relative to controls (DMSO) are presented as bar graphs. (**E**). The effective dose of NCL-1 required to inhibit SK N BE cell viability by 50% (EC_50_) at 72 h was calculated for treatment with NCL 1 alone or in combination with the indicated retinoic acid (RA) doses. The EC_50_ and the inhibitory concentration (IC_50_) values were identical. The effects of combination treatment with retinoic acid (RA) or NCL 1 alone in SK-N-BE cells on markers of differentiation are shown as bar graphs for relative NTS and MAP2 expression (qPCR) after 24 h (**F**), filamentous cytoskeletal actin by phalloidin staining in representative micrographs after 72 h (**G**) and bar graphs of total numbers of neurite-like structures visible per cell in 125 randomly selected fields after both 24 h and 72 h (**H**). The fold-changes in every treatment group were compared to those in the DMSO control (also without retinoic acid = 1). Significant differences between treatment groups were analyzed using Student’s *t*-test (* *p* < 0.05, ** *p* < 0.01, *** *p* < 0.001).

We examined the effect of NCL-1 treatment in vitro on typical tumor cell functions in neuroblastoma cells and compared these cell phenotypes with the previously reported cellular phenotype induced by siRNA-mediated KDM1A knockdown [10]. SH-EP, SK-N-BE, SK-N-AS, and IMR-5 cell lines were treated for 72 h with 40 µM NCL-1 or a DMSO solvent control before assessing the viability (MTT assay), proliferation (BrdU incorportion), cell death (ELISA), and markers for differentiation, NTS expression, and neurite outgrowth. The 40 µM concentration was chosen based on the EC_50_ that we determined in neuroblastoma cell lines to maintain a relevant proportion of living cells in order to observe biological effects. NCL-1 significantly downregulated the viability and proliferation in all four cell lines (Figure 2A). The cell death ratio detected by the cell death ELISA also increased concordantly with the drop in the viability of all the cell lines tested (Figure 2A). The NCL-1 treatment significantly upregulated NTS (a surrogate for neuronal differentiation) in all the cell lines except IMR-5, with the strongest induction occurring in SK-N-BE cells, in which expression was up to 3.5-fold higher than in the control cells (Figure 2A). Neurite outgrowth was also observed in the cells from all four cell lines that survived 72 h of NCL-1 treatment (Figure 2B). To test NCL-1 on a patient-representative model, which maintains heterogeneous genetic clonality from the originating bone marrow metastasis while growing as a mixture of adherent cells and suspended aggregates, we treated OHC-NB1 cells for 72 h with NCL 1 concentrations ranging from 5 to 180µM (or DMSO control), before assessing the viability. The NCL-1 effective dose in OHC-NB1 cells was similar to that in the four cell lines tested (EC_50_ = 132µM, Figure 2C), with the selected NCL-1 concentrations strongly inhibiting the viability in a dose-dependent manner compared to in the controls (Figure 2D), and, respectively, increasing the proportion of dead cells in the treated cultures (Figure 2D).

Since retinoic acid is known to induce neuroblastoma differentiation, we combined the NCL-1 and retinoic acid treatment to test any possible synergistic activity in SK-N-BE cells as an example. We treated the SK-N-BE cells for 72 h with 5–100 µM NCL-1 with or without 1µM or 2 µM 13-cis retinoic acid, then assessed the impact of the retinoic acid on the NCL-1-dependent EC_50_ with viability curves. Retinoic acid alone had no significant impact on the cell viability (Supplementary Figure S2), but produced an additive effect on the NCL-1-dependent reduction in viability (Figure 2E). Marker genes for differentiation (*NTS* and *MAP2*) were induced by retinoic acid and NCL-1 alone (Figure 2F), as expected. However, neither retinoic acid concentration significantly enhanced marker gene expression induced by NCL-1 treatment, indicating that the combination had no additive or synergistic effect on the differentiation-inducing capacity of either agent. The treated cells were also microscopically examined for phenotypical changes indicating differentiation, including flattening of the cellular morphology consistent with cytoskeletal changes and the formation of filamentous actin that underlies neurite outgrowth (Figure 2G). While both retinoic acid and NCL-1 treatment singly induced the concentration- and time-dependent formation of neurite-like structures as a quantitative measure of morphological differentiation, combining the two agents did not enhance their differentiation-inducing activity (Figure 2H). These results demonstrate that NCL-1 has a capacity to induce differentiation that is similar to that of retinoic acid, but that combining the agents does not enhance their differentiation-inducing capacity.

Altogether, the pharmacological inhibition of KDM1A produced a clear phenotype of reduced viability and proliferation accompanying the induction of cell death and neuronal differentiation in surviving cells. This phenotypical picture recapitulated the cellular phenotype we previously reported to be induced by KDM1A knockdown in neuroblastoma cell lines [10]. Our results indicate that NCL-1 treatment achieves a shift from a more aggressive, actively growing and undifferentiated state to a differentiated, less aggressive state or kills neuroblastoma cells in vitro.

### 3.3. NCL-1 Treatment Suppresses Xenograft Tumor Growth and Alters the Vasculature

**Figure 3:**
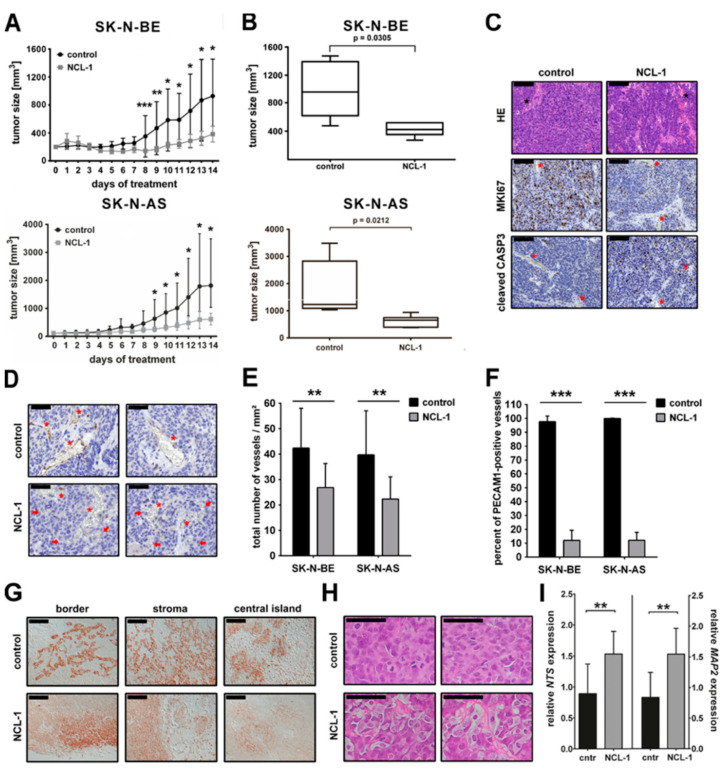
NCL-1 treatment suppresses growth of neuroblastoma xenograft tumors in mice. Xenograft tumors were generated prior to treatment from SK-N-BE and SK-N-AS cells grafted subcutaneously in NMRI nu/nu mice. Mice were intraperitoneally injected once daily with 5 mg NCL-1 in adjuvant (SK-N-AS: 10 mice, SK-N-BE: 5 mice) or adjuvant alone (SK-N-AS: 11 mice, SK-N-BE: 5 mice) as a control for 14 d. Additionally, 3 mice each engrafted with either cell line were treated for 3 d twice daily with 10 mg NCL-1. (**A**). Tumor volumes were measured using calipers, and tumor growth curves were generated using mean tumor volumes from each treatment group on each treatment day. Significant differences between treatment groups were analyzed using Student’s *t*-test (* *p* < 0.05, ** *p* < 0.01, *** *p* < 0.001). (**B**). Box and whisker plots represent final tumor volumes after 14 d of treatment. (**C**). Representative pictures are shown for SK-N-AS tumors from mice treated for 3 d. Tumor histology is shown with hematoxylin/eosin (HE) staining, and proliferating (MKI67) and apoptotic (cleaved CASP3) cells were immunohistochemically detected. Scale bars: 100 µm. (**D**). Representative pictures of PECAM1 staining in SK-N-AS tumors from mice treated for 14 d with NCL-1 or adjuvant control are shown. Scale bars: 50 µm. Vessels are indicated by asterisks and single groups of erythrocytes by arrows. (**E**). Blood vessels were counted in 8–12 random fields from PECAM1-stained sections from SK-N-BE and SK-N-AS xenograft tumors after 14 d of NCL-1 (or adjuvant control) treatment. (**F**). Numbers of vessels with endothelial cells (PECAM1-positive) were calculated for the same images as (**E**) and are shown relative to the adjuvant control. (**G**). After 14 d of treatment, hypoxic regions were stained by HIF1A enrichment in tumor border, stroma, and central islands of the tumor. Scale bars: 100 µm. (**H**). Representative histology in SK-N-BE xenografts (hematoxylin/eosin staining) after 14 d of NCL-1 or adjuvant control. Scale bars: 20 µm. (**I**). NTS and MAP2 expression (qPCR) is shown for the same SK-N-BE xenograft tumors shown in H. Significant differences between treatment groups were assessed by Student’s *t*-test (** *p* < 0.01, *** *p* < 0.001).

We next explored efficacy and response to NCL-1 in xenograft mouse models. NMRI^nu/nu^ mice harboring established tumors (50–200 mm^3^ from SK N-AS and SK-N-BE cells) were intraperitoneally injected once daily for 14 d with 200 mg/kg body weight of NCL-1 or adjuvant control. Tumor volumes in NCL-1 and control cohorts were plotted as growth curves during the treatment period to assess the efficacy in the intervention scenario. NCL-1 significantly reduced xenograft tumor growth in the treatment cohorts harboring either a cell line-derived xenograft (Figure 3A), resulting in significantly smaller tumors after 14 d of NCL-1 treatment (Figure 3B). Importantly, no adverse side effects were observed with the NCL-1 treatment as compared to the excessive side effects (including seizures) we previously observed during treatment with the MAO inhibitor transylcypromine [10]. The immunohistological assessment of the xenograft tumors treated for 3 d with 400 mg/kg body weight of NCL-1 twice daily revealed that the NCL-1 treatment reduced the proportion of proliferating (detected by MKI67 staining) cells to only 25–48% (*p* < 0.001) of that in the matched untreated control tumors, and induced apoptosis (detected by cleaved CASP3 staining) by 1.6- to 7.7-fold (*p* < 0.05) of that in the untreated control tumors (Figure 3C and Figure S3). The untreated tumors were well vascularized. Endothelial cells were unequivocally identified by PECAM1 expression (immunohistochemically) in the same sections stained with hematoxylin/eosin to show xenograft tumor histology (Figure 3C,D). The vessel density (number of vessels per mm^2^) was assessed, as was the presence of endothelial cells in the vessels (PECAM1 staining). NCL-1 treatment of mice for 14 d caused an overwhelming loss of endothelial cells in the xenograft tumors and a disruption of blood vessels, with red blood cells scattered in the tumor tissue, exemplarily shown with the SK-N-AS xenografts (Figure 3D). Treatment with NCL-1 significantly reduced the vessel density (by 33%) in both the SK-N-BE and SK-N-AS xenograft tumors (Figure 3E). Endothelial cells were present in all the vessels in the xenograft tumors from the control group, but in only 12% of the vessels in the NCL-1 treatment group (Figure 3F). In the tumors from the untreated control mice, exemplarily shown with the SK-N-AS xenografts, HIF1A was uniformly expressed at the tumor borders, stroma-associated regions lacking blood vessels, and within central islands in the xenograft tumors (Figure 3G). The NCL-1 treatment reduced HIF1A expression in all the central regions of the xenografts (where proliferation and apoptosis were also affected, Supplementary Figure S3), with expression remaining only at the xenograft borders. Our results demonstrate that NCL 1 is capable of slowing neuroblastoma cell growth and inducing apoptosis without adversely affecting the mice in an in vivo model of neuroblastoma. NCL-1 also appears to influence the tumor microenvironment by either destroying and/or preventing vascularization, implying that KDM1A might be regulating tumor angiogenesis and/or vascularization. The NCL-1 treatment produced no histological signs of differentiation in the SK-N-AS xenograft tumors, although this cell line usually does not differentiate in xenografts in our experience. NCL-1 treatment resulted in clusters of larger cells with a pale cytoplasm and less dense nuclei with prominent nucleoli (mature ganglion cells) in the SK-N-BE xenograft tumors compared to the control group (Figure 3H), clearly indicating differentiation in the NCL-1-treated SK-N-BE xenograft tumors. The NCL-1 treatment also enhanced the expression of the neuronal differentiation markers, NTS and MAP2, in the SK-N-BE xenograft tumors in comparison to the controls (Figure 3I). Our results demonstrate that NCL-1 reduces tumor xenograft growth, regulates the tumor microenvironment, and induces differentiation in vivo. The NCL-1 treatment achieves a shift from a more aggressive actively growing and undifferentiated state to a differentiated, less aggressive state or kills the neuroblastoma cells, also in vivo.

### 3.4. NCL-1 Disturbs Angiogenesis in an In Vitro Endothelial Cell Model

**Figure 4:**
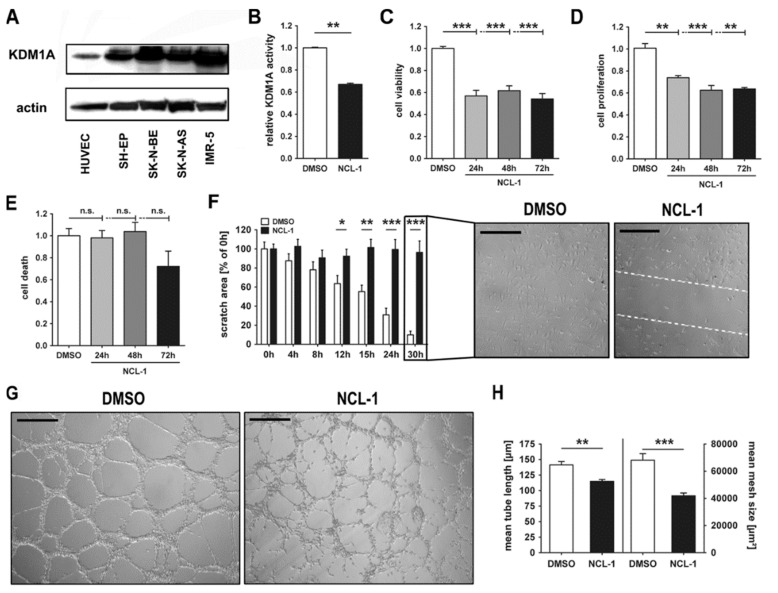
Inhibiting KDM1A in HUVEC-C cells in vitro disrupts angiogenic activities. (**A**). Whole-cell lysates were prepared from HUVEC-C and neuroblastoma cell lines in RIPA buffer with sonication to disrupt protein complexes and separated on 15% SDS-PAGE. Basal KDM1A protein expression was detected by Western blotting. HUVEC-C cells were treated with 40 µM NCL-1 or DMSO (control) for 72 h, then relative KDM1A activity was assessed using the EpiQuik™ KDM1A/LSD1 activity/inhibition assay (**B**), cell viability was assessed in MTT assays (**C**), cell proliferation was assessed in an ELISA for BrdU incorporation in DNA (**D**) and cell death was assessed in the Cell Death ELISA (**E**,**F**). Wound healing was analyzed by scratch assay, with representative pictures of scratch growing after 30 h of treatment. Scale bars: 50 µm. (**G**). Representative pictures are shown of HUVEC-C cells in tube formation assays after 8 h of treatment with NCL-1 or DMSO (control). Scale bars: 100 µm. (**H**). Mean tube length and mesh size were calculated for tubes formed after 24 h in the presence of NCL-1 or DMSO (control). Significant differences between treatment groups were assessed by Student’s *t*-test (* *p* < 0.05, ** *p* < 0.01, *** *p* < 0.001).

We further explored the effect that NCL-1 had on the xenograft tumor vasculature in an in vitro model for endothelial growth and tube formation using the HUVEC-C cell line. HUVEC-C cells express KDM1A, but to a lesser extent than in neuroblastoma cell lines (Figure 4A). The treatment of the HUVEC-C cells with NCL-1 for 72 h reduced the KDM1A activity (assessed by ELISA) by 35% (Figure 4B). NCL-1 also significantly inhibited the viability (40–45%, Figure 4C) and proliferation (Figure 4D) of the HUVEC-C cells, but did not induce apoptosis (Figure 4E) after 3 d of treatment. We examined the capacity of the HUVEC-C cells to migrate in scratch assays conducted over 30 h, and the NCL-1 treatment almost completely inhibited HUVEC-C cell migration (Figure 4F). The control HUVEC-C cells underwent tubulogenesis to create well-organized tubules in Matrigel™ after 8 h (Figure 4G). The NCL-1 treatment did not completely prevent tubulogenesis, but the tubules were less organized (Figure 4G) and the mean tube length and mesh size were significantly reduced (Figure 4H), demonstrating that proper tubulogenesis was disturbed. These results corroborate the loss of both endothelial cells and vessel integrity in the neuroblastoma xenograft tumors from the mice treated with NCL-1, and illuminate a significant anti-angiogenic effect of NCL-1 in the tumor microenvironment.

## 4. Discussion

The KDM1A small molecule inhibitor, NCL-1, reduced cell viability and proliferation and induced apoptosis in neuroblastoma cell lines with different molecular drivers of high-risk disease as well as mouse xenograft models derived from two neuroblastoma cell lines. NCL-1 also elicited an anti-angiogenic effect on the xenograft tumor microenvironment.

NCL-1 reduced the cell viability and proliferation while inducing apoptosis in both in vitro and in vivo high-risk models for neuroblastoma. This suppression of an aggressive neuroblastoma phenotype was concordant with that which we previously achieved via KDM1A knockdown in vitro and exceeded that of high-dose tranylcypromine treatment in vivo without the extreme adverse effects [10]. Increases in the dimethylation of all activating H3K4 and inhibiting H3K9 marks were dependent on the NCL-1 treatment duration and cell type in our experiments. The molecular differences among the cell backgrounds explain the variation. Since KDM1A demethylates activating or inhibiting histone marks only at specific constellations of gene promoters or enhancers in a cell [5], a net increase in the pan-dimethylation of H3K4 and H3K9 marks cannot be expected in every molecular background. The viability was reduced in six cell lines derived from different cancers by comparable NCL-1 concentrations, while H3K4 dimethylation was only slightly increased in some cell lines [21]. KDM1A also regulates non-histone proteins by demethylation, including TP53, NFkappaB, and E2F1 [34,35], which could contribute to the variable suppression of cell proliferation or induction of apoptosis in different molecular backgrounds. The impact of NCL-1 on cell viability, proliferation, and apoptotic control that we observed in the cell lines and the patient-representative OHC-NB1 model is in line with the specific inhibition of KDM1A.

NCL-1 induced neurite outgrowth in neuroblastoma cell lines that survived 72 h of treatment, as we previously observed following KDM1A knockdown [10], and upregulated known markers of neuronal differentiation. NTS is expressed in mature neurons in the brain [36] and adrenal gland [37] and in differentiated neuroblastomas [38]. MAP2 is a cytoskeletal component essential for neurite development [39]. Inhibiting KDM1A in acute myeloid leukemia cells also induces differentiation and reduces the accumulation of blast cells in vivo [5,19]. NCL-1 reduced viability and induced cell death in neuroblastoma cell lines and a patient-representative cell model. These results support the induction of programming for differentiation, which could be interpreted as a shift from an aggressive proliferative neuroblastoma phenotype to a less aggressive one.

Combining the NCL-1 treatment with retinoic acid, an agent known to induce neuroblastoma differentiation [40], reduced the cell viability and NCL-1 EC_50_. The NTS and MAP2 expression and numbers of neurite-like structures increased after NCL-1 or retinoic acid treatment alone, but appeared to act independently and not synergize. While retinoic acid has been preclinically demonstrated to act synergistically with other compounds on tumor growth [41] and differentiation [42], the newest clinical evidence indicates no clinical benefit from including retinoic acid in treatment schedules for neuroblastoma because they become resistant [43]. Our results demonstrate that KDM1A inhibition in neuroblastoma models induces differentiation independently of retinoic acid, and adding KDM1A inhibition to combination therapies independent of retinoic acid or even as a substitute for retinoic acid (to maintain a differentiation-inducing effect) may boost treatment success.

The NCL-1 treatment destroyed the blood vessel integrity and reduced the number in xenograft tumors, releasing red blood cells into the tumor tissue. We confirmed a loss of endothelial cells in the infiltrating xenograft tumor vasculature in the mice treated with NCL-1. A similar effect was reported for xenograft tumors derived from the PCai1 prostate cancer cell line in mice treated with NCL-1 [22]. Kashyap et al. demonstrated that KDM1A directly regulates VEGFA expression in prostate cancer cells [44], suggesting that the anti-angiogenic effects observed in the tumor xenografts may be occurring via KDM1A inhibition of VEGFA expression resulting from NCL-1 treatment. Thrambyrajah et al. also demonstrated that KDM1A is part of a gene group closely associated with blood vessel maintenance and remodeling [45]. We show here that NCL-1 effectively inhibited KDM1A in the HUVEC-C cells, causing not only a reduction in viability and proliferation, but almost completely preventing motility in wound healing assays and proper tubulogenesis in Matrigel™. This direct effect of NCL-1 on HUVEC-C is an anti-angiogenic mechanism beyond the mechanism of conventional angiogenesis inhibitors such as bevacizumab, which just deplete tumor cell-derived VEGF, and might explain why we do not observe normalization of the vasculature upon NCL-1 treatment. Two other anti-angiogenic agents have indeed been demonstrated to similarly affect the tumor cell phenotype and, in particular, vasculogenesis. The ER520 selective estrogen receptor modulator decreased breast and endometrial cancer cell adhesion, migration, and invasion as well as VEGF secretion and disrupted HUVEC-C tube-forming ability [46]. A specific kinase inert receptor (formerly VEGFR2) inhibitor, JFD-WS, also effectively inhibited HUVEC-C proliferation, migration, and tube formation in vitro and vasculogenesis in the chick chorioallantoic membrane [47]. Notably, both compounds also had a direct effect on HUVEC-C, as we demonstrated here with NCL-1. Our experiments showed that NCL-1 treatment also reduced HIF1A in the central regions of the xenograft tumors, where blood vessels were also lost and tumor cell proliferation was suppressed in addition to apoptosis or differentiation to ganglion cells being induced. HIF1A expression in neuroblastomas has been positively correlated with tumor aggressiveness, probably to protect against the hypoxic conditions created by their rapid growth, and hypoxia has been shown to inhibit the differentiation of neuroblastoma cell lines grown in culture or as xenografts, and may induce neuroblastoma cells to acquire a stem cell-like phenotype [48]. KDM1A also directly demethylates HIF1A at K32 [49] and K391 [50] to stabilize HIF1A against degradation. KDM1A-driven HIF1A has been associated with aggressive behavior in breast cancer cells [49], and HIF1A was shown to enhance angiogenesis, cell migration, and colony formation in the Hif1α^KA/KA^ knock-in mouse model [49]. NCL-1 most likely inhibits KDM1A-orchestrated tumor angiogenesis and/or vascularization in the tumor in addition to its effects on tumor cell renewal.

The specific and selective KDM1A inhibitor, NCL-1, may provide an avenue to reprogram aggressive neuroblastoma biology to a less proliferative and more differentiated state, and reduce hypoxia-associated pro-angiogenic pathways to disturb tumor vascularization. The effect of KDM1A inhibition on the tumor microenvironment caused by NCL-1 as well as KDM1A small molecule inhibitors that are already more advanced and currently under investigation in early clinical trials warrants further investigation.

## Data Availability

Original qPCR data presented in this study are available on request from the corresponding author.

## Supplementary Materials

The following supporting information can be downloaded at https://www.mdpi.com/article/10.3390/jcm13206081/s1: Table S1. Antibodies used in immunohistochemistry and Western blotting; Supplementary Figure S1. H3K4me2 and H3K9me2 levels increased by NCL-1 treatment; Supplementary Figure S2. NCL-1-suppressed proliferation and induced apoptosis in xenograft tumors; Supplementary Figure S3. Retinoic acid alone does not significantly change viability of SK-N-BE cells.
