# Supplementary material for: Targeting KDM1A in Neuroblastoma with NCL-1 Induces a Less Aggressive Phenotype and Suppresses Angiogenesis
Source: J Clin Med. 2024 Oct 12;13(20):6081. doi: 10.3390/jcm13206081 (PMC11508765; doi:10.3390/jcm13206081)
Supplement: Supplementary file 1 [file jcm-13-06081-s001.zip › jcm-3174411-supplementary.pdf]

Table S1. Antibodies used in immunohistochemistry (IHC) and Western blotting (WB).

**Antibody**		**Manufacturer**	**Dilution**	**Application**
**MKI67**	RM-9106-S	Thermo Fisher Scientific, Waltham, MA, USA	1:200	IHC
**Cleaved CASP3**	ASP175	Cell Signaling, Danvers, MA, USA	1:750	IHC
**PECAM1**	DIA-310-M	Dianova, Hamburg, Germany	1:25	IHC
**HIF1A**	#10006421	Cayman Chemical, Ann Arbor, MI, USA	1:20,000	IHC
**KDM1A**	#2184, clone C69G12	Cell Signaling Technology, Danvers, MA, USA	1:1,000	WB
**Dimethylated H3K4**	ab7766	Abcam, Cambridge, UK	1:1,000	WB
**Dimethylated H3K9**	ab1220	Abcam, Cambridge, UK	1:1,000	WB
**NTS**	sc-377503	Santa Cruz Biotechnology Inc., Dallas, TX, USA	1:1,000	WB
**MAP2**	#8707	Cell Signaling Technology, Danvers, MA, USA	1:1,000	WB
**Phalloidin**	8953S	Cell Signaling Technology, Danvers, MA, USA	1:20	IHC
**ACTB**	A3853, clone AC-40	Sigma-Aldrich of Merck KGaA, Darmstadt, Germany	1:4,000	WB

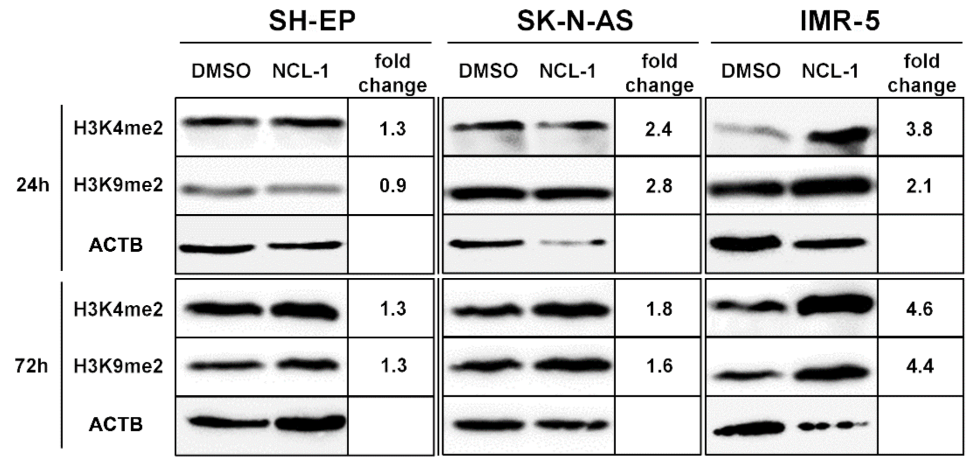

Figure S1. Semiquantitative assessment of H3K4me2 and H3K9me2 protein levels by western blot analysis. SH-EP, SK-N-AS and IMR-5 cells were treated for 24h or 72h with 40µM NCL-1 or DMSO (solvent control). Cell lysates were sonicated to extract whole proteins including histones. Dimethylation levels on lysine 4 and lysine 9 of histone 3 (H3K4me2 and H3K9me2) were detected by western blotting. ACTB served as a loading control, and the expression varied in some of the conditions investigated. Protein levels relative to DMSO control (fold change) were calculated from gel images using ImageJ software.

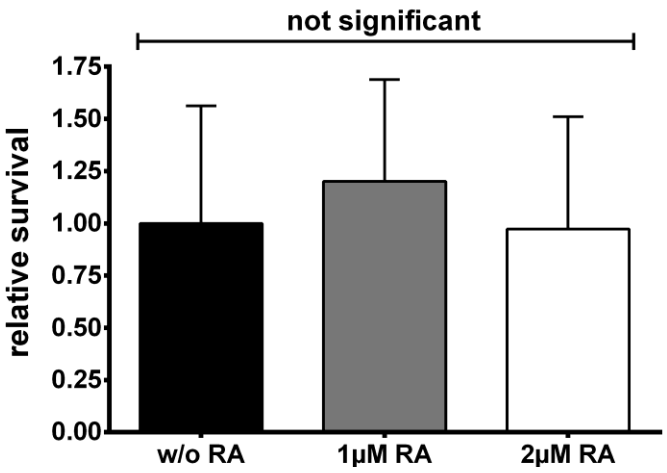

Figure S2. Retinoic acid alone does not significantly change viability in SK-N-BE cells. SK-N-BE cell line were treated without retinoic acid, with 1 µM and 2 µM. After 48h the media were refreshed. 72h after treatment cell viability was measured by CellTiter-Glo® Luminescent Cell Viability Assay and differences between treatment groups were calculated. Significant differences between treatment groups were assessed by Student's t-test.

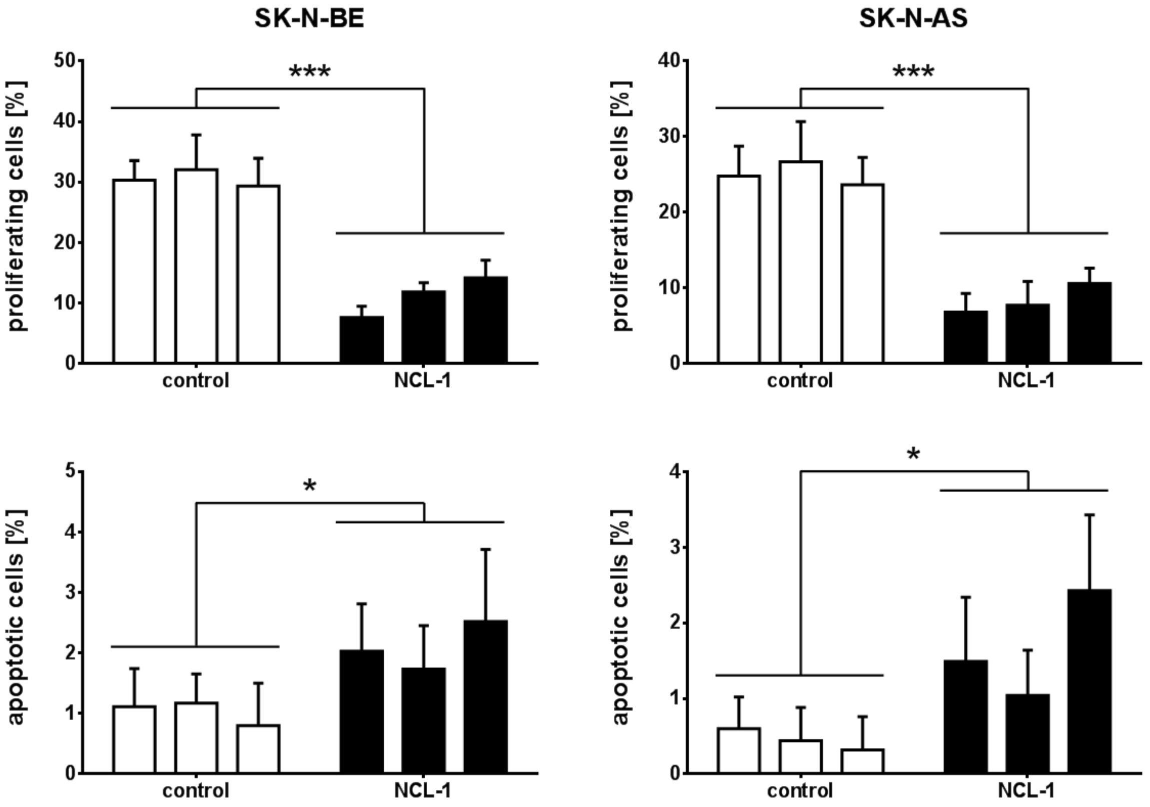

Figure S3. NCL-1 suppressed proliferation and induced apoptosis in xenograft tumors. NMRI nu/nu mice harboring tumors established from SK-N-BE (3 vs. 3 mice) or SK-N-AS (3 vs. 3 mice) cells were intraperitoneally injected for 3 days twice daily with 10mg NCL-1 or the adjuvant control. MKI67 (marker for proliferation) and cleaved CASP3 (marker for apoptosis) were immunohistochemically detected in sections of formalin-fixed, paraffin-embedded xenograft tumor sections. All cells were counted in 8-12 random fields in the central xenograft area of a single section from each tumor, and the percentages of cells staining positively for each marker in the total cell number in the field were calculated. Significant differences between treatment groups were assessed by Student's t-test (*p<0.05, ***p< 0.001).
